# A systematic review examining the relationship between cytokines and cachexia in incurable cancer

**DOI:** 10.1002/jcsm.12912

**Published:** 2022-01-25

**Authors:** D. Robert Paval, Rebekah Patton, James McDonald, Richard J.E. Skipworth, Iain J. Gallagher, Barry J. Laird, the Caledonian Cachexia Collaborative

**Affiliations:** ^1^ Faculty of Health Sciences and Sport University of Stirling Stirling UK; ^2^ St Columba's Hospice Edinburgh UK; ^3^ Ninewells Hospital Dundee UK; ^4^ Department of Clinical Surgery Royal Infirmary of Edinburgh Edinburgh UK; ^5^ Institute of Genetics and Molecular Medicine University of Edinburgh Edinburgh UK

**Keywords:** Cachexia, Cancer, Weight loss, Cytokines

## Abstract

Cancer cachexia is an unmet clinical need that affects more than 50% of patients with cancer. The systemic inflammatory response, which is mediated by a network of cytokines, has an established role in the genesis and maintenance of cancer as well as in cachexia; yet, the specific role of the cytokine milieu in cachexia requires elucidation. This systematic review aims to examine the relationship between cytokines and the cachexia syndrome in patients with incurable cancer. The databases MEDLINE, EMBASE, CINAHL, CENTRAL, PsycINFO, and Web of Science were searched for studies published between 01/01/2004 and 06/01/2020. Included studies measured cytokines and their relationship with cachexia and related symptoms/signs in adults with incurable cancer. After title screening (*n* = 5202), the abstracts (*n* = 1264) and the full‐text studies (*n* = 322) were reviewed independently by two authors. The quality assessment of the selected papers was conducted using the modified Downs and Black checklist. Overall, 1277 patients with incurable cancer and 155 healthy controls were analysed in the 17 eligible studies. The mean age of the patients was 64 ± 15 (mean ± standard deviation). Only 34% of included participants were female. The included studies were assessed as moderate‐quality to high‐quality evidence (mean quality score: 7.8; range: 5–10). A total of 31 cytokines were examined in this review, of which interleukin‐6 (IL‐6, 14 studies) and tumour necrosis factor‐α (TNF‐α, 12 studies) were the most common. The definitions of cachexia and the weight‐loss thresholds were highly variable across studies. Although the data could not be meta‐analysed due to the high degree of methodological heterogeneity, the findings were discussed in a systematic manner. IL‐6, TNF‐α, and IL‐8 were greater in cachectic patients compared with healthy individuals. Also, IL‐6 levels were higher in cachectic participants as opposed to non‐cachectic patients. Leptin, interferon‐γ, IL‐1β, IL‐10, adiponectin, and ghrelin did not demonstrate any significant difference between groups when individuals with cancer cachexia were compared against non‐cachectic patients or healthy participants. These findings suggest that a network of cytokines, commonly IL‐6, TNF‐α, and IL‐8, are associated with the development of cachexia. Yet, this relationship is not proven to be causative and future studies should opt for longitudinal designs with consistent methodological approaches, as well as adequate techniques for analysing and reporting the results.

## Introduction

Cancer cachexia is a complex syndrome characterized by the loss of skeletal muscle mass—with or without loss of fat mass—which cannot be fully reversed using standard nutritional care.^1^ This multifactorial syndrome that leads to progressive functional impairment occurs at different rates depending on the type of cancer, affects more than 50% of the patients, and accounts for 20% of cancer‐related deaths.^2^ Furthermore, it has been established that cachexia diminishes the effectiveness of anti‐cancer treatments^3^ and negatively affects patients' quality of life.^4^ To date, there is no licensed treatment and no standard of care.^5^

Cancer cachexia results from a combination of reduced energy intake, excess energy expenditure, elevated catabolism, and increased systemic inflammation.^6^ Previous research suggested that the systemic inflammatory response has a role in the progression of both cancer^7^ and cancer‐related cachexia.^6^, ^8^

Inflammation is mediated by a network of pro‐inflammatory and anti‐inflammatory cytokines that are normally in equilibrium. In the cancer state, the equilibrium is disrupted, resulting in a dysfunctional state of simultaneous immune stimulation and suppression.^9^ Cytokines operate both within the tumour micro‐environment and by interacting with other tissues in the body to generate a systemic response.^10^ Indeed, a considerable amount of evidence indicates the contribution of cytokines in cellular events that determine the initiation, promotion, invasion, and metastasis of cancer.^11^ Similarly, Fearon and colleagues^12^ highlighted that the production rate of several cytokines is associated with the prevalence of cachexia in multiple types of cancer. Even though cytokine levels were correlated with cancer and cachexia in numerous studies, the mechanisms through which these substances act upon the tumour and other body systems are not completely understood.

Multiple systematic reviews^13^, ^14^ have evaluated the relationship between cytokines and cancer. Likewise, the role of cytokines in cachexia was previously examined,^12^, ^15^, ^16^ but none of the investigations used a systematic approach to appraise the available evidence. Moreover, very few studies^17^ assessed the relationship between cytokines and cachexia in individuals suffering from incurable cancer. If the relationship between cytokines and the development of cancer cachexia was elucidated, this may identify key therapeutic targets that could be translated into clinical therapies. To date, no systematic review evaluated the relationship between cytokines and cachexia in patients with cancer. Therefore, this systematic review aimed to explore the relationship between cytokines and the cachexia syndrome (including related symptoms such as weight loss, anorexia, and reduced physical function) in people with incurable cancer.

## Methods

### Search strategy

The following databases were searched for studies published in English between 01/01/2004 and 06/01/2020: MEDLINE, EMBASE, CINAHL, CENTRAL, PsycINFO, and Web of Science. The search strategy was verified by a subject librarian and included (but was not limited to) the following terms: cytokine, interleukin, interferon AND cancer, metastasis, neoplasm AND cachexia, weight loss, anorexia (Supporting Information, *Document* S1).

### Inclusion and exclusion criteria

Eligible studies met the following criteria: adults (>18 years old); diagnosed with incurable cancer, defined as metastatic cancer or locally advanced cancer treated with palliative intent; measured the level of one or more cytokines; and assessed at least one symptom and/or sign associated with cachexia. Studies examining all primary cancer types were included to ensure that as much information as possible regarding cytokines and cachexia was retrieved. Diagnosis of cachexia was based on the criteria reported by primary authors rather than any specific definition, allowing the inclusion of studies conducted before 2011, the year when the Fearon definition was published.^1^ This ensures that as many studies as possible were included, regardless of the definitions or the weight‐loss thresholds used to diagnose cachexia.

Studies were excluded if the participants were cancer survivors or being treated with curable intent. Additionally, the studies were not considered for inclusion if patients' symptoms were attributed directly to a form of therapy or medication. Although no criterion regarding the study design was imposed, the current review did not consider case studies, animal models, protocols, or conference abstracts.

### Study selection and quality assessment

**Figure 1:**
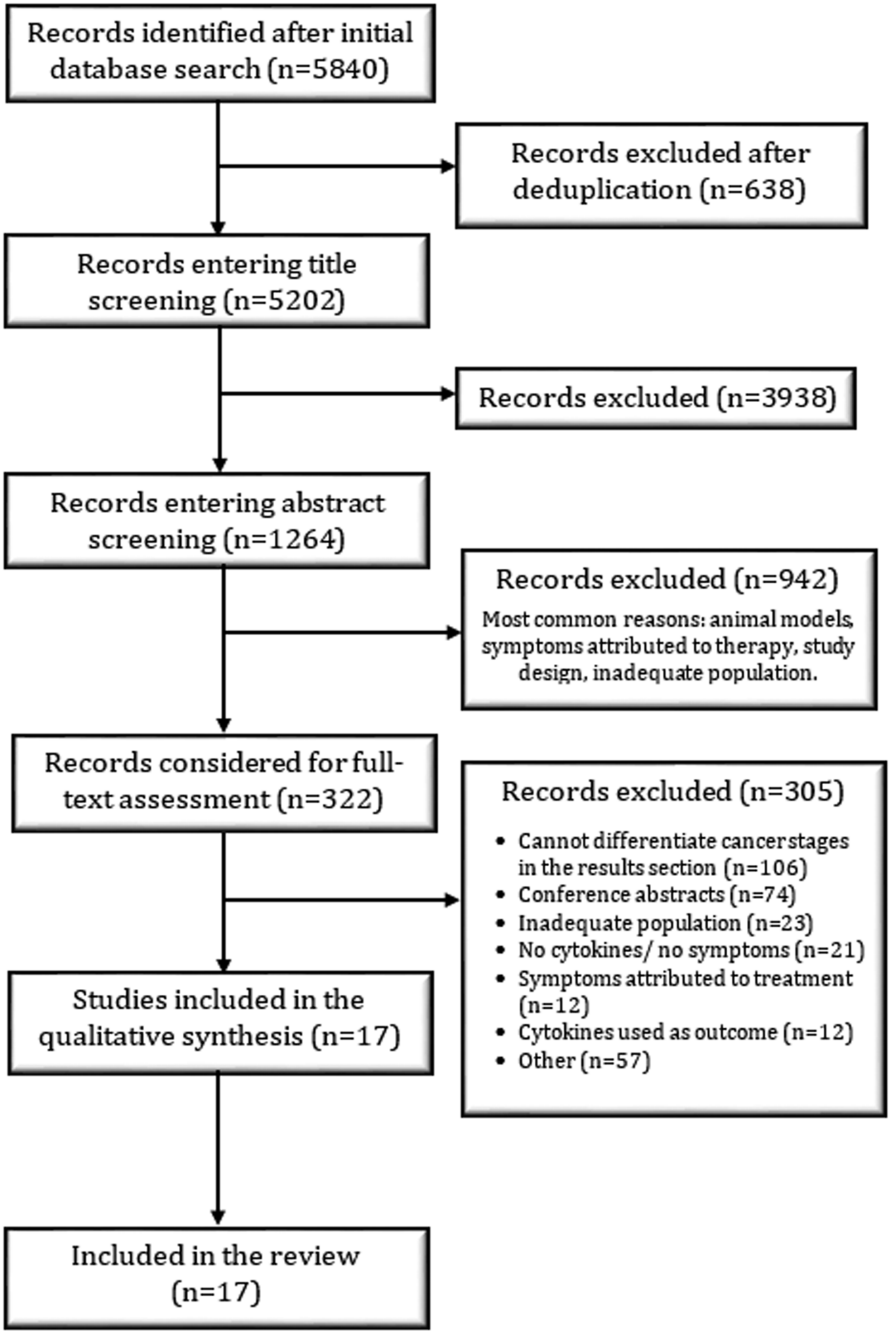
Flow diagram of the study selection protocol.

*Figure* 1 highlights the Preferred Reporting Items for Systematic Reviews and Meta‐Analyses (PRISMA) flow diagram of study selection. The titles of the studies were screened independently by R. P. using a conservative approach—whenever the title did not provide enough information, the study was included in the next selection phase. Abstract screening was conducted by D. R. P. and R. P. in a similar manner, and the studies identified as relevant were accepted for full‐text assessment. Following full‐text assessment (D. R. P. and R. P.), the quality of the included studies was appraised by D. R. P., R. P., and J. M. using the modified Downs and Black (MDB) checklist.^18^ Studies were rated on a scale from 0 to 10 using standardized criteria, and the quality of the evidence was classified as follows: 0–4 low quality, 5–7 moderate quality, and 8–10 high quality.

### Data extraction, management, and analysis

A specifically designed collection form was used to systematically capture all the relevant information from the eligible studies. Where studies measured cytokines at multiple time points (2/17 studies), only baseline data were included. No statistical analyses were conducted due to the great level of heterogeneity in study design and data reporting identified between the included studies. Thus, the findings are presented in a descriptive manner, highlighting similarities and discrepancies as well as strengths against weaknesses from the available literature. Lastly, no ethical approval was required for this systematic review.

## Results

### Study characteristics

A total of 5202 studies were identified after removing the duplicates from the database search (*Figure* 1). After evaluating the titles, 1264 studies were included in the abstract screening phase, of which 322 were selected for full‐text screening. At the end of the study selection process, 17 studies met the inclusion criteria of this systematic review. *Table* 1 summarizes the main characteristics of the eligible studies. Overall, 1277 patients with incurable cancer and 155 healthy controls from 13 different middle‐income and high‐income countries^36^ were recruited from both inpatient and outpatient settings. The mean age of the patients was 64 ± 15 (mean ± standard deviation), with female participants making up only a third (34%) of the sample. The most common types of cancers were lung and pancreatic cancer, although various other types such as colorectal, breast, gastric, or oesophageal cancer were evaluated.

**Table 1:** Characteristics of the included studies

Author (year)	Country	Participants	Cancer type	Cytokines	Blood collection method	Assay method	Sensitivity reported	MDB score
Fortunati et al. (2007)^19^	Italy	33 patients 23 controls	Lung cancer (non‐small cell, small cell, and adenocarcinoma)	TNF‐α, IL‐6	Morning, overnight fast	ELISA	Yes	6
Grim‐Stieger et al. (2008)^20^	Austria	61 patients	Breast, colorectal, lung, pancreatic, gastric, and renal cancer	TNF‐α, IL‐6	Morning, overnight fast	ELISA	No	8
Takahashi et al. (2009)^21^	Japan	16 patients 10 controls	Oesophageal, gastric, colorectal cancer	TNF‐α, IL‐6, IFN‐γ, IL‐1Ra, leptin, ghrelin	Morning, overnight fast	ELISA	No	5
Gioulbasanis et al. (2011)^22^	Greece	115 patients	Lung cancer (non‐small cell and small cell)	Leptin, adiponectin, ghrelin	N/R	RIA	Yes	8
Scheede‐Bergdahl et al. (2012)^23^	Canada	83 patients	Gastrointestinal and non‐small cell lung cancer	IL‐1β, IL‐6, IL‐8, TNF‐α	Morning, overnight fast	Bio‐Plex cytokine assay	No	7
Op den Kamp et al. (2013)^24^	Netherlands	26 patients 22 controls	Non‐small cell lung cancer	IL‐6, IL‐8, IL‐10, TNF‐α, IFN‐γ	N/R	Multiplex antibody assay	Yes	7
Fujiwara et al. (2014)^25^	Japan	21 patients	Pancreatic cancer	IL‐6, TNF‐α, leptin	Morning, overnight fast	RIA and ELISA	Yes	10
Lu et al. (2014)^26^	China	110 patients	Oesophageal squamous cell carcinoma	MIC‐1	N/R	ELISA	No	7
Bilir et al. (2015)^27^	Turkey	46 patients 34 controls	Gastroesophageal, pancreatic, lung, colorectal, ovarian, breast, and laryngeal cancer	IL‐1α, IL‐1β, IL‐6, TNF‐α	Morning, overnight fast	ELISA	No	8
Srdic et al. (2016)^28^	Croatia	100 patients	Advanced non‐small cell lung cancer	IL‐6	N/R	ECLIA	No	8
Penafuerte et al. (2016)^29^	Canada	122 patients	Head, neck, breast, upper gastrointestinal, lung, hepatobiliary, prostate, and colorectal cancer	IL‐1α, IL‐1β, IL‐3, IL‐4, IL‐5, IL‐6, IL‐8, IL‐10, IL‐12, IL‐15, IL‐18, IFN‐γ, MCP‐1, TNF‐α, leptin, ghrelin, adiponectin, TRAIL, TGF‐β1	N/R	Bio‐Plex cytokine assay	No	9
Lerner et al. (2016)^30^	USA	218 patients	Lung and pancreatic cancer	IL‐1, IL‐2, IL‐4, IL‐5, IL‐6, IL‐7, IL‐8, IL‐9, IL‐10, IL‐12, IL‐13, IL‐17, IFN‐γ, GDF‐15, MCP‐1, IP‐10	N/R	Bio‐Plex cytokine assay	No	9
Bye et al. (2016)^31^	Norway	20 patients 40 controls	Pancreatic cancer	IL‐6, IL‐10, TNF‐α, adiponectin, leptin, IFN‐γ	Non‐fasting	ELISA	Yes	8
Fogelman et al. (2017)^32^	USA	89 patients 6 controls	Pancreatic cancer	IL‐1β, IL‐6, IL‐8, TNF‐α, leptin, adiponectin, ghrelin	N/R	N/R	N/R	7
Demiray et al. (2017)^33^	Turkey	67 patients 20 controls	Non‐small cell lung cancer	Leptin, resistin	Morning, overnight fast	ELISA	No	8
Murton et al. (2017)^34^	UK	4 patients 4 controls	Advanced non‐small cell lung cancer	IL‐6, TNF‐α	Morning, overnight fast	ELISA	No	6
Hou et al. (2018)^35^	Taiwan	146 patients	Pancreatic cancer	IL‐1β, IL‐6, IL‐8, TNF‐α	N/R	ELISA	No	10

ECLIA, electrochemiluminescence immunoassay; ELISA, enzyme‐linked immunosorbent assay; MDB, modified Downs and Black; N/R, not reported; RIA, radioimmunoassay.

The majority of the studies (15/17) measured and reported cytokine levels at a single time point. One study^25^ measured intra‐day cytokine variation. In this instance, the morning measurements were used in this systematic review as they were taken after an overnight fast. One study^31^ measured patients' cytokine levels at enrolment and every 4 weeks until death. This study reported baseline and endpoint data. The baseline measurements were extracted and used in the present review as the endpoint data were not reported separately for cachectic and non‐cachectic patients. The mean quality score of the papers from the current review was 7.8 (range 5–10), indicating that the studies incorporated evidence of moderate to high quality (*Table* 1). Although 6 studies were of moderate quality and 11 were of high quality, several methodological weaknesses were persistent across study reports. The majority of the included studies were marked down as they failed to meet various methodological norms that had an impact on both internal and external validity. Most commonly, the data were not fully reported for all the measured cytokines—some studies specified central tendency values and measures of dispersion only for statistically significant relationships, while other papers only reported *P* values (*Table* 2). Additionally, several studies did not accurately describe participants' selection criteria and/or the sample collection methodology.

**Table 2:** Main findings of the included studies

Authors (year)	Variable measured	Grouping criteria	Findings	Data
Fortunati et al. (2007)^19^	Cachexia	Cachexia defined as more than 5% weight loss in the previous 6 months.	TNF‐α levels were greater in CC patients compared with NC individuals (*P* < 0.05) and controls (*P* < 0.01). IL‐6 was greater in CC patients compared with controls (*P* < 0.01) but did not significantly differ from NC patients (*P* > 0.05).	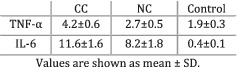

Grim‐Stieger et al. (2008)^20^	Weight loss	Evaluated whether participants suffered any weight loss since diagnosis or in the past 3 months.	No significant correlations found between TNF‐α and WL since diagnosis (*P* = 0.19) or during the last 3 months (*P* = 0.11). No significant correlation found between IL‐6 and WL since diagnosis (*P* = 0.13) or during the last 3 months (*P* = 0.12).	Only *P* values.
Takahashi et al. (2009)^21^	Cachexia	No definition.	CC patients expressed greater levels of TNF‐α, IL‐6, IL‐1Ra (*P* < 0.01), and ghrelin (*P* = 0.04) compared with healthy participants. No difference in IFN‐γ was observed between groups (*P* = 0.27), while leptin was significantly higher in healthy controls (*P* = 0.02).	Only *P* values.

Gioulbasanis et al. (2011)^22^	Weight loss and nutritional sufficiency	Patients divided into Group A—nutritional sufficiency (15% lost more than 5% body weight), Group B—risk of malnutrition (63% lost weight), and Group C—malnourished (83% lost weight).	The mean levels of leptin were significantly higher (*P* < 0.01) in Group A compared with Group B and Group C. Less adiponectin (*P* = 0.06) was detected in Group A compared with Group B and Group C. Ghrelin levels did not significantly differ (*P* > 0.05) between Groups A, B, and C.	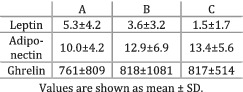

Scheede‐Bergdahl et al. (2012)^23^	Weight loss and sarcopenia	Participants grouped based on the degree of weight loss 6 months prior enrolment—more or less than 5% weight loss. The presence of sarcopenia assessed by calculating the appendicular lean mass index (Baumgartner, 2000).^37^	The study compared high versus low levels of cytokines. Higher levels of IL‐1β and TNF‐α were significantly (*P* < 0.01) associated with the presence of more than 5% weight loss. The levels of IL‐6 and IL‐8 could not significantly predict (*P* > 0.05) weight loss. Similarly, IL‐1β and TNF‐α were positively associated (*P* < 0.05) with the presence of sarcopenia, while a trend existed for both IL‐6 (*P* = 0.06) and IL‐8 (*P* = 0.09).	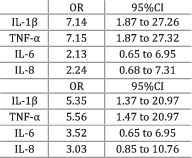

Op den Kamp et al. (2013)^24^	Cachexia	Participants grouped based on the 2011 consensus definition of cancer cachexia.	Significantly (*P* < 0.05) higher levels of IL‐6 and IL‐8 were observed in the plasma of CC patients compared with individuals with PC and controls. IFN‐γ was significantly higher (*P* < 0.05) in controls compared with PC patients. The levels of TNF‐α and IL‐10 did not differ between groups.	Only *P* values.

Fujiwara et al. (2014)^25^	Cachexia	Cachexia defined as ECOG PS 1 to 4, Grade 1 to 4 anorexia, and more than 10% weight loss over the past 6 months.	IL‐6 (*P* = 0.35), TNF‐α (*P* = 0.27), and leptin (*P* = 0.27) levels did not differ between CC and NC patients.	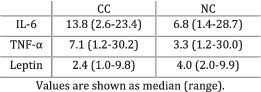

Lu et al. (2014)^26^	Weight loss	Participants divided based on the degree of weight loss before chemotherapy—more or less than 5%.	MIC‐1 levels were significantly higher (*P* = 0.01) in patients with >5% weight loss compared with those with ≤5% weight loss.	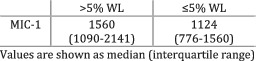

Bilir et al. (2015)^27^	Cachexia	Cachexia—BMI < 20, weight loss during treatment, or weight loss of more than 5% prior to illness in the past 6 months and continuing in the last few months. Refractory cachexia—patients unresponsive to treatment with a life expectancy lower than 3 months and reduced performance status.	IL‐1α (*P* = 0.03), IL‐6 (*P* < 0.01), and TNF‐α (*P* < 0.01) were higher in people suffering from CC compared with controls. IL‐1β (*P* = 0.6) did not differ between groups. IL‐1α was higher in individuals with CC compared with patients with RC (*P* = 0.02). IL‐1β was greater in patients with RC compared with individuals with CC (*P* = 0.01). IL‐6 (*P* = 0.70) and TNF‐α (*P* = 0.12) did not differ between groups.	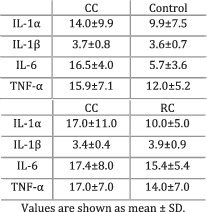

Srdic et al. (2016)^28^	Cachexia	Participants grouped based on the 2011 consensus definition of cancer cachexia.	Patients with CC had significantly higher levels of IL‐6 compared with patients with NC (*P* = 0.04).	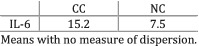

Penafuerte et al. (2016)^29^	Cachexia	Participants grouped based on the 2011 consensus definition of cancer cachexia.	TGF‐β1: patients with CC (*P* < 0.01) and PC (*P* = 0.04) expressed higher levels compared with NC patients; no difference between PC and CC (*P* > 0.05). IL‐8: Patients with CC showed greater levels than individuals with PC (*P* < 0.01) and NC (*P* < 0.01); no difference between PC and NC (*P* > 0.05). IL‐6: greater in patients with CC compared with NC (*P* < 0.01); no difference between PC and CC and between PC and NC (*P* > 0.05). TRAIL: levels higher in patients with CC compared with NC individuals; no difference between PC and CC and between PC and NC (*P* > 0.05).	Only *P* values. Data were not reported for all cytokines.

Lerner et al. (2016)^30^	Weight loss	Participants divided based on the degree of weight loss—more than 5%, between 0% and 5%, and no weight loss.	GDF‐15 was greater in patients with >5% WL (*P* < 0.01) and with ≤5% WL (*P* < 0.01) compared with individuals with no WL. IL‐12 levels were greater in individuals with >5% WL compared with both ≤5% WL (*P* = 0.03) and no WL (*P* < 0.01). IL‐10 levels were higher in patients with >5% WL compared with both ≤5% WL (*P* = 0.05) and no WL (*P* = 0.05). IL‐7 was greater in participants with >5% WL compared with both ≤5% WL (*P* = 0.08) and no WL (*P* = 0.06). Participants with >5% WL showed greater IL‐6 (*P* = 0.06) and IL‐2 (*P* = 0.07) levels as opposed to patients with no WL. The levels of IL‐13 (*P* = 0.04), IL‐8 (*P* = 0.06), and IL‐9 (*P* = 0.08) were higher in participants with >5% WL compared with individuals with ≤5% WL. All other relationships showed greater, non‐significant *P* values.	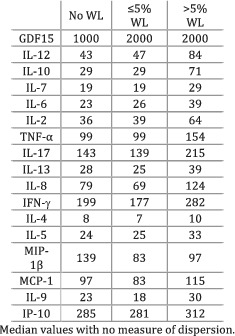

Bye et al. (2016)^31^	Cachexia	Participants grouped based on the 2011 consensus definition of cancer cachexia and on the modified Glasgow Prognostic Score (mGPS)	CC and NC were determined according to the 2011 consensus, and no difference in cytokine levels was observed between the groups (*P* > 0.05). IL‐6 was greater in CC patients compared with NC individuals, when the disease was assessed according to mGPS (*P* < 0.01). The other cytokines did not differ between groups (*P* > 0.05)	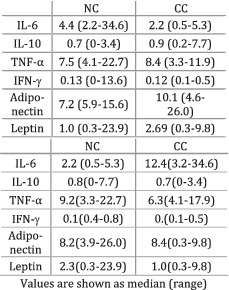

Fogelman et al. (2017)^32^	Weight loss	The participants in the weight loss group had either 10% weight loss or died at 60 days after baseline. The non‐weight loss group failed to meet any of the aforementioned criteria.	IL‐1β: levels were greater in the control group compared with both no WL and WL (*P* = 0.07); the levels were higher in the no WL group compared with WL (*P* = 0.03). IL‐6: levels were smaller in the control group compared with both WL (*P* < 0.01) and no WL (*P* < 0.01); the levels were higher in the WL group compared with no WL (*P* = 0.03). TNF‐α: levels were smaller in the control group compared with both WL (*P* < 0.01) and no WL (*P* < 0.01); levels were higher in the WL group compared with no WL (*P* = 0.03). IL‐8: levels were smaller in the control group compared with both WL (*P* < 0.01) and no WL (*P* < 0.01); no significant differences observed between the WL and no WL groups (*P* > 0.05). All other relationships showed greater, non‐significant *P* values.	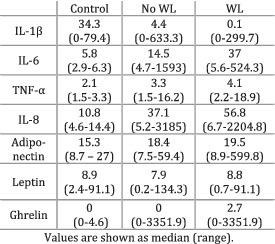

Demiray et al. (2017)^33^	Weight loss	Weight loss at the time of diagnosis defined as more than 10% weight loss within the past 6 months.	The levels of leptin (*P* = 0.44) and resistin (*P* = 0.54) did not differ between patients with and without WL.	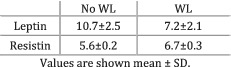

Murton et al. (2017)^34^	Cachexia	Participants grouped based on the 2011 consensus definition of cancer cachexia.	Cachectic individuals showed higher levels of IL‐6 (*P* < 0.05) and TNF‐α (*P* = 0.06) compared with healthy controls.	Only *P* values.

Hou et al. (2018)^35^	Cachexia	Participants grouped based on the 2011 consensus definition of cancer cachexia.	There was a positive (*P* = 0.03) relationship between IL‐8 and WL. Also, a positive relationship (*P* = 0.07) was observed between IL‐6 and WL. The other correlations were less strong and showed greater *P* values. IL‐8 levels were greater in CC compared with NC (*P* = 0.01). IL‐1β (*P* = 0.95), IL‐6 (*P* = 0.16), and TNF‐α (*P* = 0.84) levels did not differ between CC and NC patients.	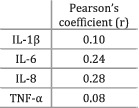

BMI, body mass index; CC, cancer cachexia; CI, confidence interval; CTR, control; ECOG PS, Eastern Cooperative Oncology Group Performance Status; NC, cancer non‐cachexia; OR, odds ratio; PC, pre‐cachexia; RC, refractory cachexia; SD, standard deviation; WL, weight loss.

A methodological characteristic that played a pivotal role in the included studies was the timing of blood sampling as previous research^38^, ^39^ suggested that cytokine levels show intra‐day variation. Only eight studies indicated that blood was collected in the morning after an overnight fast, while the others provided relatively vague information about this matter (i.e. before chemotherapy, using standard methods) or failed to specify the period of the day when blood sampling was performed (*Table* 1). Furthermore, almost all studies (16/17) reported the assay used to quantify cytokine levels, but only five reported the sensitivity of the assay. The enzyme‐linked immunosorbent assay (ELISA) was the most used quantification method, whereas other validated methods such as the electrochemiluminescence immunoassay (ECLIA) and the radioimmunoassay (RIA) were used in some studies.

### Main findings

*Table* 2 highlights the main findings of the included studies as well as relevant data and grouping criteria. The studies included in the current review analysed the relationship between cytokine levels and cachexia or the degree of weight loss experienced by cancer patients. A great level of variation was observed between the definitions of cachexia and the weight‐loss thresholds used across the studies to classify and group participants. A third of the included studies defined cachexia as suggested by Fearon and colleagues,^1^ while several studies referred to cachexia as a syndrome that implies losing more than 5%^19^ or 10%^25^ body weight. Furthermore, some authors did not use the term ‘cachexia’ but instead classified the participants according to the amount of weight lost during a period of 3–6 months before the study. The criteria according to which participants were grouped are not homogenous across studies. Although the data could not be subject to a meta‐analysis due to the methodological differences, a systematic summary of the findings is subsequently described and discussed.

A total of 31 different (adipo)cytokines were measured across the 17 studies included in the present review (*Table* 1). The most frequently analysed cytokines were interleukin (IL)‐6 (14), tumour necrosis factor‐α (TNF‐α) (12), leptin (7), IL‐8 (6), IL‐1β and interferon‐γ (IFN‐γ) (5), and IL‐10, ghrelin, and adiponectin (4).

The majority (11/14) of the studies analysing IL‐6 indicated the presence of a relationship between high levels of IL‐6 and cachexia or weight loss. Cachectic (weight‐losing) patients showed significantly more IL‐6 compared with healthy control groups in six out of six studies. When cachectic (weight‐losing) individuals were compared with non‐cachectic (weight‐stable) cancer patients, five out of eight studies indicated that the levels of IL‐6 were significantly higher in cachectic participants. A study that compared pre‐cachectic patients against those with cancer cachexia observed greater levels of IL‐6 in the latter group,^24^ while two other studies did not find any differences between pre‐cachexia and cachexia.^29^, ^30^ Furthermore, two studies^20^, ^23^ did not find any statistically significant relationship between IL‐6 and weight loss, while Hou *et al*. (2018) indicated the presence of a medium association (*r* = 0.24, *P* = 0.07). Interestingly, Scheede‐Bergdahl and colleagues^23^ indicated that higher IL‐6 levels were positively associated with the presence of sarcopenia. Thus, the evidence suggests higher IL‐6 expression in cachectic patients compared with non‐cachectic counterparts and healthy individuals.

Another cytokine showing a relationship with the presence of cancer cachexia and weight loss was TNF‐α. The levels of TNF‐α were significantly higher in cachectic (weight‐losing) patients compared with healthy controls in five out of six studies. The sixth study^30^ also found a greater concentration of TNF‐α in the cachectic group, but the difference was not statistically significant. Only two out of six papers indicated that cachectic (weight‐losing) patients expressed more TNF‐α than non‐cachectic (weight‐stable) counterparts, while the other studies did not find any statistically significant differences between groups. Likewise, no difference was observed between pre‐cachectic and cachectic patients, while two other studies did not find any significant correlation between TNF‐α and weight loss. Therefore, TNF‐α levels are elevated in cachectic patients compared with healthy controls, while no significant distinction was noticed between weight‐stable and weight‐losing cancer patients.

Similar to the aforementioned cytokines, but with fewer studies to support the findings, IL‐8 levels were overall higher in cachectic (weight‐losing) patients. The studies that compared healthy controls against individuals with cancer cachexia (*n* = 2) reported that the levels of IL‐8 were significantly higher in the diseased group. Additionally, two out of three studies that examined IL‐8 levels in cachectic (weight‐losing) and non‐cachectic (weight‐stable) cancer patients found that IL‐8 was increased in cachectic participants. Lastly, individuals with cancer cachexia had more IL‐8 compared with pre‐cachectic patients in both studies that examined this comparison. Overall, IL‐8 showed increased levels in participants with cancer cachexia and weight loss compared with non‐cachectic, pre‐cachectic, and healthy groups, but the strength of these observations is limited given the small number of studies analysing this cytokine.

Leptin, IFN‐γ, IL‐1β, IL‐10, adiponectin, and ghrelin did not demonstrate any significant difference between groups when cachectic (weight‐losing) patients were compared against non‐cachectic (weight‐stable) counterparts or healthy participants (*Table* 2). However, a study worth mentioning was conducted by Scheede‐Bergdahl and colleagues^23^ who observed that higher levels of IL‐1β, as opposed to low IL‐1β concentrations, were significantly associated with the presence of more than 5% weight loss [odds ratio (OR) = 7.14, *P* < 0.01] and sarcopenia (OR = 5.35, *P* < 0.05). The other cytokines listed in *Table* 1 are not discussed because they were analysed by two or fewer studies and not enough information was available.

## Discussion

### Main findings

The aim of the current review was to examine the relationship between cytokines and the cachexia syndrome (including related symptoms such as weight loss, anorexia, and reduced physical function) in people with incurable cancer irrespective of tumour type. Overall, IL‐6, TNF‐α, and IL‐8 were present in greater concentrations in patients losing weight as opposed to healthy individuals. Leptin, IFN‐γ, IL‐1β, IL‐10, adiponectin, and ghrelin were also evaluated, but no relationship was observed between the cytokines' circulating levels and the degree of weight loss. Moreover, the definitions of cachexia and the weight‐loss thresholds used across the studies to categorize participants were heterogeneous and a more consistent approach should be adopted for future studies.

The levels of circulating IL‐6 were elevated in weight‐losing and cachectic patients compared with healthy controls in all studies that analysed this cytokine. Furthermore, more than half of the studies that compared cachectic and weight‐losing patients with non‐cachectic or weight‐stable counterparts indicated the presence of higher IL‐6 concentrations in cachectic individuals. The direction of these relationships was also observed by other research and it has been previously suggested that IL‐6 is a central regulator of the progression of cancer and cancer‐associated cachexia.^40^, ^41^, ^42^ Several studies examined the effect of IL‐6 inhibitors on cachexia. Clazakizumab, an anti‐IL‐6 antibody, was tested in patients with non‐small cell lung cancer and improved cachexia and anaemia in phase I and II trials.^43^ Despite the fact that the drug seemed well tolerated, there is no phase III trial ongoing. Furthermore, various case reports^44^, ^45^ and animal models^46^ indicated that tocilizumab might ameliorate cancer‐associated cachexia. Often used in patients with rheumatoid arthritis, tocilizumab was associated with increased weight and body mass index in a recent systematic review.^47^ Although the previously mentioned reports suggest a potential positive effect of tocilizumab, no clinical trials are currently examining its effect on cancer cachexia. To conclude, assessing the circulating levels of IL‐6 could be a useful method of monitoring the development of cancer cachexia and future trials should aim to integrate the cytokine in the multifactorial management of this disorder.

Circulating TNF‐α was expressed in higher concentrations in cachectic and weight‐losing patients as opposed to healthy individuals. There was no difference in TNF‐α when cachectic and weight‐losing patients were compared with non‐cachectic and weight‐stable patients. The available literature highlights the role of TNF‐α as a key mediator of cachexia given the cytokine's ability to activate nuclear factor‐κB, one of the main pathways that determine skeletal muscle atrophy.^48^, ^49^ Various studies focused on analysing the effectiveness of TNF‐α inhibitors such as etanercept and infliximab.^50^, ^51^, ^52^ In a cohort of patients with incurable cancer, etanercept only produced a small level of weight gain and failed to treat cachexia.^50^ Similarly, pancreatic cancer patients receiving infliximab gained an insignificant amount of weight compared with counterparts receiving a placebo. Another trial analysing the effectiveness of OHR/AVR118, an agent targeting both IL‐6 and TNF‐α, indicated that cancer patients with cachexia patients improved anorexia, strength, and dyspepsia.^53^ This finding reinforces the idea that not one, but multiple cytokines could be responsible for the onset and progression of cancer cachexia and a multimodal approach is required in the management of this disorder.

The majority of the studies analysing IL‐8 indicated that the cytokine's expression was greater in patients with cancer cachexia and weight loss compared with non‐cachectic, weight‐losing, pre‐cachectic, and healthy individuals. Although the strength of this observation is limited given the small number of papers examining this cytokine, future research might evaluate the direction of the relationship between IL‐8 and cachexia because this matter was not thoroughly explained by the available literature. Furthermore, none of the other cytokines analysed in the current review showed any relationship with the amount of weight lost by patients. However, previous research linked cytokines such as IL‐1α,^54^ IL‐1β,^55^ and IFN‐γ^56^ with the occurrence and development of weight loss. Overall, there is not enough evidence available regarding the previously mentioned cytokines to reach a definitive conclusion and future studies should aim to explore this knowledge gap.

### Inconsistencies in grouping criteria

The studies included in the current review used distinct methods of defining cachexia and various weight‐loss thresholds to group participants (*Table* 2). Some studies used the consensus definition from 2011^1^ or the modified Glasgow Prognostic Score^57^ to assess and diagnose cachexia. Multiple studies^26^, ^30^ used a 5% weight loss limit as the main grouping criterion and only discussed patients' weight without referring to cachexia as a disorder. Interestingly, various papers classified participants using weight‐loss thresholds that appeared to be chosen arbitrarily (i.e. 10%), while others used cachexia definitions that were not validated by previous literature (*Table* 2). Thus, the results could not be meta‐analysed due to the lack of a consistent method of grouping participants. The current review presented findings in a descriptive manner, giving a useful indication of the trajectory of the available evidence. However, conducting a meta‐analysis would provide a more precise and reliable summary of the included studies and should allow an effective comparison between them.^58^ Consequently, practitioners could make well‐informed decisions based on high‐quality evidence with a lower risk of bias^59^ and this would have a positive impact on patients' treatment and quality of life. Future studies should adhere to definitions and thresholds that are already established by the literature in order to promote uniformity and consensus in the field of cancer cachexia. Otherwise, any novel method for defining and assessing cachexia should be accompanied by a thorough rationale.

### Limitations and directions for future research

Most of the studies analysed in this review had a cross‐sectional design and do not allow the inference of a causal relationship between cytokines and cachexia. A limitation of the present findings is that only two studies reported multiple cytokine measurements and only the baseline data were used in the current review. Future work in this area should assess cytokine levels longitudinally to fully elucidate their effect on the cachexia phenotype. Moreover, the relationship between cytokines and cachexia was examined in all primary tumour types. Although this may be considered a limitation because cachexia is less common in some cancers, failing to include all primary tumour types means that minimal data would be available and important studies might be omitted.

Numerous papers were excluded from the present review as the data of patients with early and advanced forms of cancer were combined in the analysis. Although relevant evidence might have been left out of this study, the information about individuals with incurable malignancies could not be differentiated from the data of patients with operable forms of cancer. Additionally, the assay used to measure cytokine levels is an important methodological factor and it was reported in all but one investigation. However, less than a third of the studies indicated the sensitivity of the assay and, thus, the validity of the results that failed to consider this parameter was low.

Several other errors were observed in the statistical analysis of the results and in the methods used to report findings. In the present systematic review, the available body of literature could not be meta‐analysed due to the high degree of methodological heterogeneity as well as the lack of transparency and failure to meet basic standards of data reporting. Specifically, five studies^20^, ^21^, ^24^, ^29^, ^34^ only reported *P* values, while two studies^28^, ^30^ did not report any measure of dispersion (i.e. standard deviation and interquartile range). Several studies examined multiple cytokines and only displayed data for statistically significant relationships. The use of these practices in the literature leads to biased reporting and inflation of type I errors in systematic reviews. One study^35^ examined the correlation coefficient between cytokines and weight loss, while the other nine studies used different methods of reporting data (i.e. measure of central tendency, dispersion, or effect sizes). The remaining studies have major inconsistencies in grouping criteria. Only one study^31^ used the Fearon definition,^1^ while another classified participants based on nutritional sufficiency.^22^ Two studies^25^, ^33^ grouped participants based on a 10% weight‐loss threshold in the last 6 months, while another study^32^ used the same threshold but measured at 60 days prior enrolment. The last four studies^19^, ^23^, ^26^, ^27^ grouped patients based on a 5% weight loss limit. Yet, not even these studies could be meta‐analysed because they measured different cytokines and use dissimilar methods of reporting data (mean and standard deviation, OR and confidence interval, as well as median and interquartile range). To enable meta‐analyses in the future, consensus on cachexia definition, detailed reporting, as well as the standardization of cytokines measured and assays used would be optimal.

Although this review provided useful information, it also highlighted areas where research could be optimized. Future studies should be longitudinal, with an extensive characterization of the cachexia phenotype (including loss of lean mass/weight, patient‐reported outcomes of anorexia, fatigue, and quality of life, physical activity, and other measures of function), allowing a better understanding of the relationship between cytokines and the phenotype. Additionally, future studies should incorporate surrogate markers of the inflammatory response such as acute‐phase proteins (i.e. C‐reactive protein and serum amyloid A) and also cytokine receptors (i.e. sIL‐6R, sIL‐2R, IL1‐R1, IL1‐R2, TNF‐R1, and TNF‐R2). Adding these markers as a complementary measurement would generate a more accurate overview of the inflammatory state and of the cascade of immune events underlying cancer cachexia. As previously mentioned, increasing homogeneity in study design should be a priority for future research. This can be achieved by grouping participants according to established criteria such as the Fearon definition^1^ or the modified Glasgow Prognostic Score.^57^ Most importantly, regardless of the study design chosen by researchers, it is crucial to describe the methodology and the results in a transparent manner. Specifically, all measured variables should be reported and not only the significant results (complete datasets can be added as supplementary material to increase a manuscript's reliability); authors should go beyond *P* values and must report data using central tendency values or effect sizes alongside measures of dispersion; the blood collection methods, the type of assay used to measure biomarkers, and the sensitivity of the assay should be described in the methods section.

## Conclusions

A relationship between cytokines, cachexia, and weight loss was observed in the current review. The levels of IL‐6 and TNF‐α were greater in cachectic patients compared with healthy individuals. A similar result was obtained for IL‐8, but fewer studies supported the finding. IL‐6 was the only cytokine expressed in higher concentrations in cachectic participants compared with non‐cachectic cancer patients. The other cytokines analysed did not show any notable relationship with cachexia or the amount of weight lost by cancer patients. These findings indicate that a network of cytokines including IL‐6, TNF‐α, and IL‐8 are associated with the development of cancer cachexia. An index created from multiple cytokines might serve as a ‘biomarker’ that could be used to analyse the onset and progression of cancer cachexia. However, this relationship is not causal and future work should opt for longitudinal designs with consistent methodological approaches, as well as adequate mechanisms of analysing and reporting results.

## Conflict of interest

None declared.

## Supporting information


**Data S1.** Supporting Information.
