# Supplementary material for: A systematic review examining the relationship between cytokines and cachexia in incurable cancer
Source: J Cachexia Sarcopenia Muscle. 2022 Jan 25;13(2):824–38. doi: 10.1002/jcsm.12912 (PMC8977958; doi:10.1002/jcsm.12912)
Supplement: Supplementary file 1 — Data S1. Supporting Information. [file JCSM-13-824-s001.docx]

**Supporting Information Document S1**

**Detailed search strategy**

*Ovid Medline(R) and Epub Ahead of Print, In-process & Other Non-Indexed Citations and Daily*

1. exp cancer/
2. neoplasm.ab,ti.
3. metastasis$.ab,ti.
4. 1 or 2 or 3
5. exp cytokines/
6. exp Inflammation/
7. exp interferon/
8. tumo$r necrosis factor.ab,ti.
9. tnf.ab,ti.
10. exp interleukin/
11. 5 or 6 or 7 or 8 or 9 or 10
12. symoptom*.ab,ti.kw.
13. depress*.ab,ti.kw.
14. nausea.ab,ti.kw.
15. fatigue.ab,ti.kw.
16. sleep.ab,ti.kw.
17. quality of life.ab,ti.kw.
18. anxiety.ab,ti.kw.
19. pain.ab,ti.kw.
20. cachexia.ab,ti.kw.
21. weight loss.ab,ti.kw.
22. anorexia.ab,ti.kw.
23. 12 or 13 or 14 or 15 or 16 or 17 or 18 or 18 or 20 or 21 or 22
24. 4 and 11 and 23
25. limit 24 to (humans and yr=”2004-Current”)

*Ovid EMBASE 1980 to 2019 Week 49*

1. exp cancer/
2. neoplasm.ab,ti.
3. metastasis$.ab,ti.
4. 1 or 2 or 3
5. exp cytokines/
6. exp Inflammation/
7. exp interferon/
8. tumo$r necrosis factor.ab,ti.
9. tnf.ab,ti.
10. exp interleukin/
11. 5 or 6 or 7 or 8 or 9 or 10
12. symoptom*.ab,ti.kw.
13. depress*.ab,ti.kw.
14. nausea.ab,ti.kw.
15. fatigue.ab,ti.kw.
16. sleep.ab,ti.kw.
17. quality of life.ab,ti.kw.
18. anxiety.ab,ti.kw.
19. pain.ab,ti.kw.
20. cachexia.ab,ti.kw.
21. weight loss.ab,ti.kw.
22. anorexia.ab,ti.kw.
23. 12 or 13 or 14 or 15 or 16 or 17 or 18 or 18 or 20 or 21 or 22
24. 4 and 11 and 23
25. limit 24 to (humans and yr=”2004-Current”)

*CINAHL Complete on EBSCO host*

S1 MJ cancer

S2 TI neoplasm OR AB neoplasm

S3 TI metastasis OR AB metastasis

S4 S1 OR S2 OR S3

S5 MH cytokines

S6 MJ interleukin

S7 MH inflammation

S8 MJ interferon

S9 TI tumor necrosis factor OR AB tumor necrosis factor

S10 TI tnf OR AB tnf

S11 S5 OR S6 OR S7 OR S8 OR S9 OR S10

S12 TI symptom OR AB symptom OR SU symptom

S13 TI depression OR AB depression OR SU depression

S14 TI fatigue OR AB fatigue OR SU fatigue

S15 TI sleep OR AB sleep OR SU sleep

S16 TI quality of life OR AB quality of life OR SU quality of life

S17 TI anxiety OR AB anxiety OR SU anxiety

S18 TI pain OR AB pain OR SU pain

S19 TI cachexia OR AB cachexia OR SU cachexia

S20 TI weight loss OR AB weight loss OR SU weight loss

S21 TI anorexia OR AB anorexia OR SU anorexia

S22 TI nausea OR AB nausea OR SU nausea

S23 S12 OR S13 OR S14 OR S15 OR S16 OR S17 OR S18 OR S19 OR S20 OR S21 OR S22

S24 S4 AND S11 AND S23

*PsycINFO on EBSOC host*

S1 MJ cancer

S2 TI neoplasm OR AB neoplasm

S3 TI metastasis OR AB metastasis

S4 S1 OR S2 OR S3

S5 MJ cytokines

S6 MJ interleukin

S7 MJ inflammation

S8 MJ interferon

S9 TI tumor necrosis factor OR AB tumor necrosis factor

S10 TI tnf OR AB tnf

S11 S5 OR S6 OR S7 OR S8 OR S9 OR S10

S12 TI symptom OR AB symptom OR SU symptom

S13 TI depression OR AB depression OR SU depression

S14 TI fatigue OR AB fatigue OR SU fatigue

S15 TI sleep OR AB sleep OR SU sleep

S16 TI quality of life OR AB quality of life OR SU quality of life

S17 TI anxiety OR AB anxiety OR SU anxiety

S18 TI pain OR AB pain OR SU pain

S19 TI cachexia OR AB cachexia OR SU cachexia

S20 TI weight loss OR AB weight loss OR SU weight loss

S21 TI anorexia OR AB anorexia OR SU anorexia

S22 TI nausea OR AB nausea OR SU nausea

S23 S12 OR S13 OR S14 OR S15 OR S16 OR S17 OR S18 OR S19 OR S20 OR S21 OR S22

S24 S4 AND S11 AND S23

*Central – Cochrane Library*

1. MeSH descriptor: [Cytokines] explode all trees
2. MeSH descriptor: [Inflammation] this term only
3. MeSH descriptor: [Interferons] explode all trees
4. MeSH descriptor: [Interleukins] explode all trees
5. MeSH descriptor: [Tumor Necrosis Factors] in all MeSH products
6. MeSH descriptor: [Acute-Phase Proteins] explode all trees
7. (cytokine*):ti,ab,kw
8. (interleukin*):ti,ab,kw
9. #1 or #2 or #3 or #4 or #5 or #6 or #7 or #8
10. MeSH descriptor: [Neoplasms] explode all trees
11. (“Cancer”):ti,ab,kw
12. MeSH descriptor: [Neoplasm Metastasis] explode all trees
13. #10 or #11 or #12
14. symptom:ti,ab,kw
15. depression:ti,ab,kw
16. nausea:ti,ab,kw
17. fatigue:ti,ab,kw
18. sleep:ti,ab,kw
19. quality of life:ti,ab,kw
20. anxiety:ti,ab,kw
21. pain:ti,ab,kw
22. cachexia:ti,ab,kw
23. weight loss:ti,ab,kw
24. anorexia:ti,ab,kw
25. #14 or #15 or #16 or #17 or #18 or #19 or #20 or #21 or #22 or #23 or #24
26. #9 and #13 and #25 with Publication Year from 2004 to 2019, in Trials

*Web of Science – Core collection*

1. TS=(cancer)
2. TS=(neoplasm)
3. TS=(metastasis)
4. #1 or #2 or #3
5. TS=(cytokine*)
6. TS=(inflammation)
7. TS=(interferon)
8. TS=(tumor necrosis factor)
9. TS=(tnf)
10. TS=(interleukin)
11. #5 or #6 or #7 or #8 or #9 or #10
12. TI=(symptom)
13. TI=(depression)
14. TI=(nausea)
15. TI=(fatigue)
16. TI=(sleep)
17. TI=(quality of life)
18. TI=(anxiety)
19. TI=(pain)
20. TI=(cachexia)
21. TI=(weight loss)
22. TI=(anorexia)
23. #12 or #13 or #14 or #15 or #16 or #17 or #18 or #19 or #20 or #21 or #22
24. #4 and #11 and #23
